# Moderate alkali-thermophilic ethanologenesis by locally isolated *Bacillus licheniformis* from Pakistan employing sugarcane bagasse: a comparative aspect of aseptic and non-aseptic fermentations

**DOI:** 10.1186/s13068-017-0785-1

**Published:** 2017-04-24

**Authors:** Qurat-ul-Ain Ahmad, Shang-Tian Yang, Maleeha Manzoor, Javed Iqbal Qazi

**Affiliations:** 1 Department of Zoology, University of the Punjab, Lahore, Pakistan; 2 Department of Chemical & Biomolecular Engineering, Ohio State University, Columbus, OH USA; 3 Government College University, Faisalabad, Pakistan

**Keywords:** 2G ethanologenesis, Lignocellulosic ethanol fermentation, Fibrous-bed bioreactor, Gas stripping, Central composite design, Taguchi orthogonal array

## Abstract

**Background:**

Biofuels obtained from first-generation (1G) sugars-starch streams have been proven unsustainable as their constant consumption is not only significantly costly for commercial scale production systems, but it could potentially lead to problems associated with extortionate food items for human usage. In this regard, biofuels’ production in alkali-thermophilic environs from second-generation (2G) bio-waste would not only be markedly feasible, but these extreme conditions might be able to sustain aseptic fermentations without spending much for sterilization.

**Results:**

Present investigation deals with the valuation of ethanologenic potential of locally isolated moderate alkali-thermophilic fermentative bacterium, *Bacillus licheniformis* KU886221 employing sugarcane cane bagasse (SCB) as substrate. A standard 2-factor central composite response surface design was used to estimate the optimized cellulolytic and hemicellulolytic enzymatic hydrolysis of SCB into maximum fermentable sugars. After elucidation of optimized levels of fermentation factors affecting ethanol fermentation using Taguchi OA L27 (3^13) experimental design, free cell batch culture was carried out in bench-scale stirred-tank bioreactor for ethanol fermentation. Succeeding fermentation modifications included subsequent substrate addition, immobilized cells fibrous-bed bioreactor (FBB) incorporation to the basic setup, and performance of in situ gas stripping for attaining improved ethanol yield. Highest ethanol yield of 1.1406 mol ethanol/mol of equivalent sugars consumed was obtained when gas stripping was performed during fed-batch fermentation involving FBB under aseptic conditions. Despite the fact that under non-aseptic conditions, 30.5% lesser ethanol was formed, still, reduced yield might be considered influential as it saved the cost of sterilization for ethanol production.

**Conclusion:**

Effectual utilization of low-priced abundantly available lignocellulosic waste sugarcane bagasse under non-aseptic moderate alkali-thermophilic fermentation conditions as directed in this study has appeared very promising for large-scale cost-effective bioethanol generation processes.

**Electronic supplementary material:**

The online version of this article (doi:10.1186/s13068-017-0785-1) contains supplementary material, which is available to authorized users.

## Background

Energy is regarded as the lifeline of economic amelioration. Bio-conversion of different organic wastes to bioenergy fuels via microbial fermentations is being looked upon seriously all over the world due to its sustainable productive potential. Consequently, valorization of second-generation (2G) lignocellulosic biomass (LCB) through bio-fermentation employing extremophiles is the strategic tool leading to sustainable process development for biofuels’ generation. Biofuels are principally derived from plant biomass through microbial fermentations [1].

2G biofuels productions primarily address the renewable, non-food competing biomass-to-biofuels conversions that might mitigate further environmental deterioration, already reached to an alarmingly significant level in the form of pollution and global warming [2].

LCB (primarily comprises of cellulose, hemicellulose, and lignin) is first degraded into long-chain polysaccharides and then into their corresponding monosaccharides [3–5].

Several pretreatment methodologies for the separation of cellulose and hemicelluloses from lignin have been projected including acidic methods [6], hydrothermal processing [7], mild alkaline methods [6], oxidative methods [8], ionic liquid method [9], and chemical pulping methods [10, 11]. Unlike acid and hydrothermal pretreatments, alkaline pretreatment results not only in reduced hemicellulose solubilization but also lesser fermentation inhibitors production [6]. It results in increased enzymatic convertibility of accessible cellulose and hemicellulose into fermentable sugars, thus overcoming the biomass recalcitrance that poses itself as a prime impediment for LCB saccharification and subsequent fermentation into ethanol at large [12, 13].

Considerable efforts are being done worldwide to develop cost-competitive processes for biofuels production [1]. In this regard, sugarcane bagasse (SCB) represents itself as potential valuable 2G fuels’ feed, adequately rich in energy for fermentative processes. By exploiting extremophiles in bio-fermentations, the risk of microbial contamination(s) decreases, and thus, aseptic environment may be maintained with fewer efforts [14, 15]. Hence, the use of fermentative extremophiles may prove feasible and cost-competitive while developing biofuels production processes under non-aseptic conditions in near future [16].

Acetone–butanol–ethanol (ABE) productions from sugar-starch substrates are well renowned. Research is being targeted to develop ABE fermentation processes associated with high yields, titers, and productivities utilizing 2G agro-industrial wastes [17, 18].

This study mainly focuses on the application of locally isolated alkali-thermophilic bacterium *Bacillus licheniformis* KU886221 for ethanol production employing SCB as substrate. It is anticipated that bio-fermentations utilizing agro-industrial LCB, when establish, will ultimately lead to efficient waste reduction, low CO_2_ emission, and cost-effective production of high-grade liquid fuels as ethanol.

## Methods

### Isolation, screening, and characterization of alkali-thermophilic ethanologenic bacteria

Alkali-thermophilic bacteria were isolated from the soil sampled from the vicinity of hot water effluent near Balkasar oil refinery, Chakwal, Pakistan, where the mud temperature was 50–55 °C with pH ranging 9–10. The samples were incubated anaerobically at 45 °C (pH 9) for 5 days in fermentation medium FM1 containing g/L, glucose; 20, KH_2_PO_4_; 1, MgSO_4_; 0.1, CaCl_2_·2H_2_O; 0.1, yeast extract; 0.5 and peptone; 15. Further enrichment of the cultures was done in the same medium substituting 2% glucose with 5% sugarcane bagasse (SCB). On the bases of conspicuous growth on solidified fermentation medium, 34 isolates were further processed for screening of fermentative ethanologens. The initial selection of the eight ethanologenic bacteria was accomplished through the improved method of Snell and Snell [19].

For quantifying their ethanologenic potential, 50 mL of medium in 150 mL serum bottle was made for each isolate and purged with nitrogen for 8 min to remove oxygen. Then immediately, the serum bottles were firmly capped with rubber stopper and sealed with aluminum rings. These bottles were autoclaved for 30 min at 121 °C. After cooling, the bottles were inoculated using 5% inoculum in 5 mL syringe under aseptic conditions. Gas chromatograph (Shimadzu GC-2014) used was equipped with flame ionization detector (FID) and a 30 m fused silica column (0.25 m film thickness and 0.25 mm ID, Stabilwax-DA). Nitrogen was used as carrier gas at 1.47 mL/min (linear velocity: 35 cm/s). For analysis, 1 mL of sample was taken in Eppendorf and centrifuged at 13,000 rpm for 10 min. Clear supernatant was transferred to another Eppendorf and diluted 20 times before injecting to GC with a GC internal standard buffer solution (0.5 g/L isobutanol, 1% phosphoric acid and 0.1 g/L isobutyric acid (used for acidification). The column temperature was held at 80 °C for 3 min, raised to 150 °C at a rate of 30 °C/min, and held at 150 °C for 3.7 min. Both the injector and detector were set at 250 °C. Then, with the help of GC auto-injector (AOC-20i Shimadzu), 1 μL sample was injected for analysis. The bacterial isolate ML-07 was found to produce highest ethanol and selected to further study the fermentation kinetics for bench-scale bioethanol fermentation.

Isolate ML-07 was characterized genotypically following its 16S rRNA gene sequencing. The DNA sequence was assembled with BioEdit Sequence Alignment Editor (version 7.2.5). Homology finding of the isolate was accomplished using BLAST [20]. 16S rRNA gene sequence was finally submitted to GenBank for procuring accession numbers.

### Study of ethanologenesis employing different sugars and nitrogen sources in fermentation medium

Potential of the ethanologenic bacterium to ferment different sugars (glucose, xylose, mannose, galactose, arabinose, and cellulose) was evaluated by incorporating them as sole carbon source in FM1, whereas the potential of corn steep liquor as nitrogen source was evaluated by replacing 20 g of CSL with peptone + yeast extract in FM1 medium. Ethanol production by isolation of ML-07 was measured using gas chromatograph after 3 days of incubation at 45 °C (pH 9).

### SCB hydrolysis

SCB provided by Ohio State University, “Columbus Ohio”, was hydrolyzed chemically as well as enzymatically.

#### Chemical hydrolysis of SCB

SCB were pretreated differently with varying concentrations of pretreatment chemicals (sulfuric acid, phosphoric acid, hydrochloric acid, and sodium hydroxide). For every pretreatment, the solid-to-liquid ratio was kept 1:12 and autoclaved for 30 min. The cooled pretreated biomasses were immediately separated into liquid and solid fractions by vacuum filtration using Whatman No. 1 filter paper. The solid fraction was re-suspended in water and filtered three times to neutralize the pH and dried at 60 °C until the dried biomass weight became constant.

The compositions of the raw and pretreated SCB were determined by a standard analysis procedure, which was modified by the National Renewable Energy Laboratory (NREL) analytic methods [21]. The acid(s) and alkaline pretreatments were compared with respect to biomass dissociation into sugars, acids, and phenolic compounds. These components were quantified by high-performance liquid chromatography (HPLC) equipped with Bio-Rad HPX-87H column at 65 °C and a refractive index detector (Shimadzu RID-10A). The eluent (mobile phase) was H_2_SO_4_ (0.5 mN) at 0.6 mL/min.

#### Enzymatic hydrolysis of SCB

The raw and pretreated SCB were suspended in 50 mM citrate buffer (pH 5) in separate containers in solid-to-liquid ratio 1:8 and then hydrolyzed enzymatically with commercially available Cellic Ctec 2 + Cellic Htec 2 Novozymes (13.5 + 1.5 mg/g). The enzymatic hydrolysis was done under sterilized conditions in 250-mL Erlenmeyer flasks at constant agitation (150 RPM) and 50 °C incubation temperature for 72 h. The hydrolysates were analyzed for sugar contents using HPLC.

### Ethanologenesis employing pretreated SCB with/without enzymatic hydrolysis

The effect of acid/alkali pretreatment on lignocellulosic biomass and successive enzymatic hydrolysis on ethanol fermentation was studied for selection of pretreatment methods (in their optimum concentrations) of SCB for maximum ethanol production. Differently pretreated SCB (in their optimum concentrations for maximum sugars’ production) following with/without enzymatic hydrolysis were substituted with carbon source in fermentation medium. Ethanol produced by isolate ML-07 up to 72 h of fermentation was quantified by GC, whereas sugars consumption was measured using HPLC.

### Optimization of enzymatic hydrolysis by CCD

The response surface methodology (RSM) was applied to study the effect of Cellic Ctec 2 and Cellic Htec 2 Novozymes on the hydrolysis of alkaline pretreated SCB. The experiments were carried out according to central composite (2^2^) non-factorial surface design in one block. The response was total sugar contents (glucose and xylose) of pretreated SCB hydrolysate. The Statistica software (version 6.0) from Stat Soft, Inc. was used to design and statistically analyze the experiment. For the design model, five different coded levels for each variable were used: 1.41, −1, 0 (center) and +1, +1.41. The designs of all 11 experiments were performed in duplicates and the data produced were the mean value of the results. The second-order polynomial model was constructed using the CCD.

### Experimental design for optimization of fermentation using Taguchi orthogonal array (OA)

Taguchi OA method was used to design the experiment for optimization of fermentation parameters for ethanol production. For this purpose, experiment was designed by Taguchi OA L27 (3^13) of Design Expert software (version 8) to optimize six factors at three levels. The factors included three medium components including alkali treated SCB hydrolysate (SCBH) derived from alkaline pretreated SCB (after enzymatic hydrolysis), corn steep liquor (CSL), sodium chloride (NaCl), incubation temperature, incubation pH, and incubation time. Taguchi OA L27 represents the 27 experimental trials/runs, while each run consisted of a certain blend of levels to which the factors were fixed. These combinations were obtained by crossing of the factors with particular levels. These 27 runs trials were performed in duplicates using 150 mL serum bottles and the averages of obtained results were statistically investigated for the analysis of variance (ANOVA).

### Fermentation in bioreactor

**Fig. 1:**
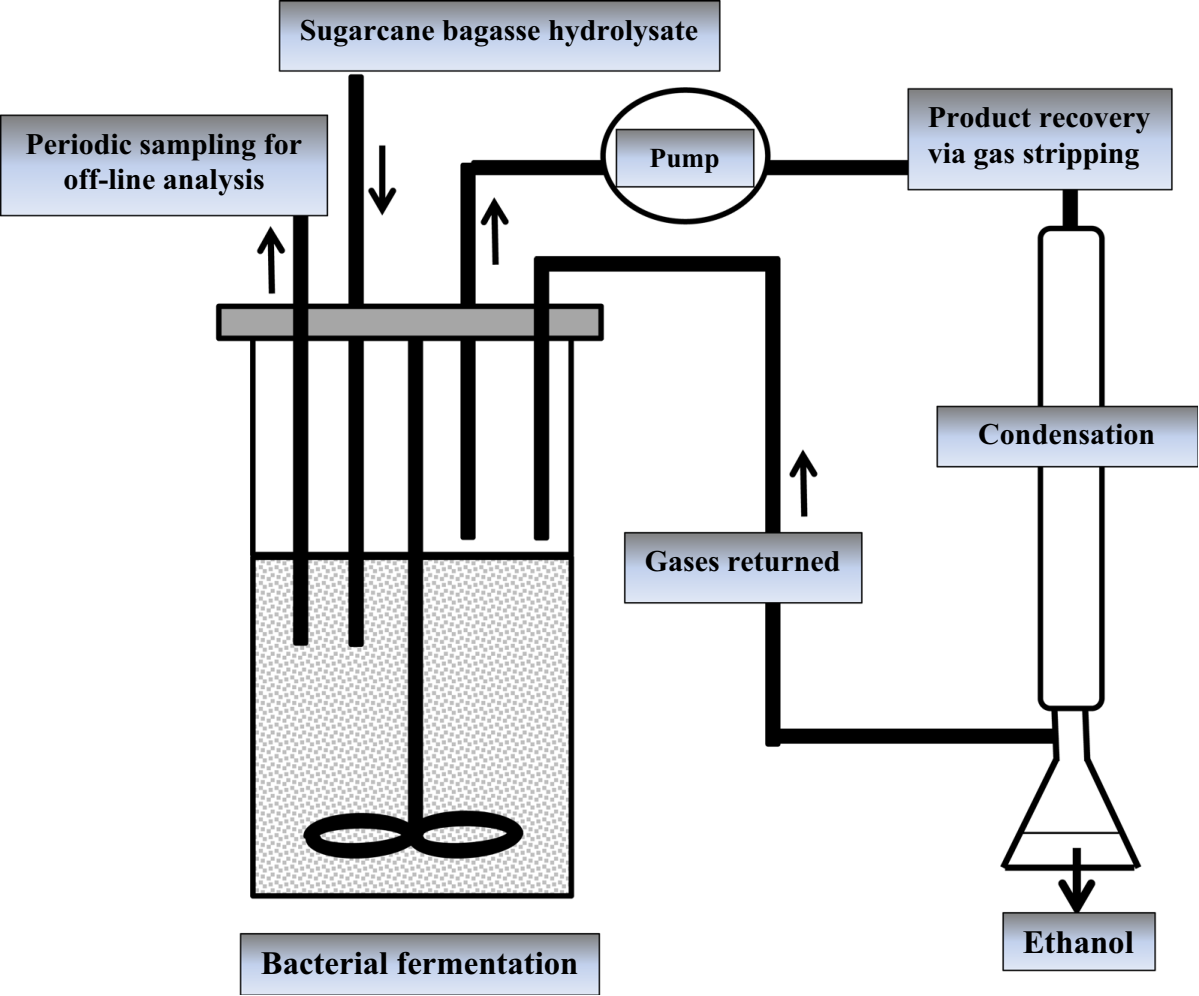
Simplified process design for second-generation ethanol fermentation from biomass and downstream product recovery by in situ gas stripping

The optimized conditions of fermentation factors were further scrutinized in bench-scale stirred-tank bioreactor under controlled pH, temperature, and agitation (140 RPM) in aseptic environment. FM2 fermentation medium was prepared by substituting the optimized amounts of three medium components elucidated by Taguchi OA experimental design to study the fermentation kinetics in bioreactor. The setup of fermentor is shown in Fig. 1.

The seed culture was prepared in 150 mL serum bottle under optimized conditions of fermentation for 48 h and was inoculated in 1.5 L fermentation medium in bioreactor. The samples were taken periodically after every 12 h and analyzed for ethanol fermentation with GC and substrate utilization with HPLC and for O.D. with spectrophotometer (Shimadzu, Columbia, MD, UV-16-1).

#### Immobilized-cell fermentation in fibrous-bed bioreactor (FBB)

**Fig. 2:**
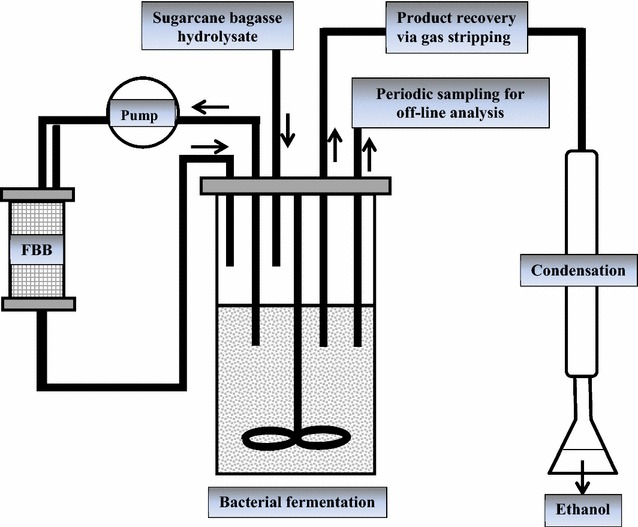
Simplified process design for second-generation ethanol fermentation involving fibrous-bed bioreactor (FBB) from biomass and downstream product recovery by in situ gas stripping

To study the effect of cell immobilization on fermentation kinetics, FBB was attached with the stirred-tank bioreactor (Fig. 2) at 24 h when the optical density of the cell suspension was above 0.6. FBB was a glass vessel whose column was packed with spirally intricate fibrous matrix to immobilize the cells [22, 23].

The fermentation broth containing the cells was pumped into the FBB top from the fermentor and sent out from the bottom of FBB back into the fermentor. This allowed the broth cells to attach and immobilize on the fibrous matrix of FBB. Fermentation kinetics of fed-batch bioreactor involving FBB was studied at regular intervals of 12 h until the culture stopped consuming substrate/producing ethanol.

#### Downstream product recovery via gas stripping

For ethanol recovery, gas stripping was done at the end of fermentation for 3 h via recycling the fermentation gases produced during fermentation through culture medium. Gas stripping was done through the fermentor to selectively strip the fermentation products (acetone, butanol, and ethanol) from fermentor broth. Detailed description for the setup and operation of the integrated system has been given previously [24, 25]. The condensate was analyzed with GC for stripped fermentation products.

#### Effect of ethanol removal on fermentation

To diminish the effect of ethanol on productivity, gas stripping was also done during the fermentation (at 60 h) when the ethanol concentration was above 10.0 g/L. The ethanol yield, titer, and productivity along with substrate utilization were noted during fed-batch fermentation involving FBB and gas stripping. The condensate obtained as a result of 6 h of gas stripping (3 h gas stripping at 60 h of fermentation and then 3 h of gas stripping at the end of fermentation (at 120 h) was analyzed with GC.

#### Bioethanol production under non-aseptic conditions

Fermentation kinetics of the fed-batch fermentation involving FBB was finally studied under non-aseptic conditions. The ethanol yield, titer, and productivity along with substrate utilization during fermentation under non-aseptic conditions were noted. Gas stripping was done at the end of fermentation for 3 h. The condensate was analyzed with GC for stripped fermentation products.

## Results

### Selection of ethanologenic alkali-thermophilic bacteria

Of the 34 initially selected isolates based on their ethanol production, as assessed by Snell and Snell’s modified method or ethanol quantification, eight were selected for further study. Ethanol production of isolate ML-07 was found to be significantly greater (*P* < 0.05) accounting 3.65 g/L, and therefore, it was selected for further study focusing on fermentation kinetics for highest production of ethanol. The gas chromatography analysis of the fermentation products also revealed that isolate ML-07 was the best ethanologenic bacteria producing 2.32 g/L ethanol amongst all other isolates.

### Genotypic identification of bacterial isolate

The isolate ML-07 was characterized on the basis of 16 s RNA gene sequencing. It was accordingly identified as a mild alkali-thermophilic strain of *B. licheniformis.* 16S rRNA gene sequences were submitted to GenBank for procuring with the accession number KU886221 assigned to this isolate.

### Ethanologenesis employing different sugars and nitrogen sources in fermentation medium

The investigation on ethanologenic potential of *B. licheniformis* KU886221 to ferment different sugars directed that the strain has the ability to produce 5.6, 4.4, and 2.23 g/L ethanol employing glucose, xylose, and cellulose as carbon source, respectively, in the fermentation medium. Moreover, substitution of corn steep liquor in FM1 medium for peptone + yeast extract improved ethanol production to 5.75 from 5.57 g/L in the FM1 medium.

### Chemical hydrolysis of sugarcane bagasse

**Fig. 3:**
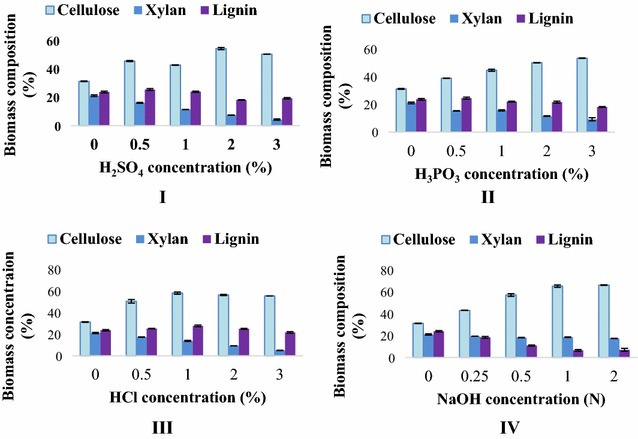
Analysis of sugarcane bagasse constituents resulted from different chemical pretreatments at various concentrations (**I**–**IV**)

Compositions of control and pretreated bagasse are shown in Fig. 3. Analysis shows that pretreatment with 2% H_2_SO_4_, 3% H_3_PO_4_, and 1% HCl resulted in comparable cellulose enrichment and xylan removal (*P* < 0.05), whereas with 1 N NaOH, statistically highest yields of cellulose and xylan were obtained.

### Enzymatic hydrolysis of sugarcane bagasse

**Fig. 4:**
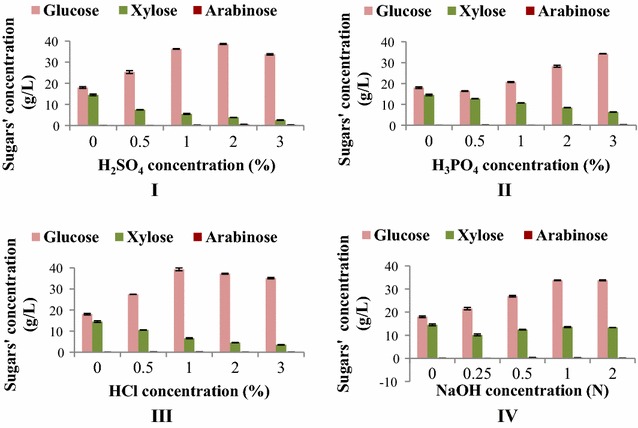
Sugars’ concentrations after enzymatic hydrolysis following different pretreatment methods using SCB 100 g/L (**I**–**IV**)

The pretreated SCB were further hydrolyzed enzymatically and studied for release of sugar contents (Fig. 4). After enzymatic hydrolysis, SCB pretreated with 2% H_2_SO_4_, 3% H_3_PO_4_, and 1% HCl released significantly increased levels of glucose, whereas SCB pretreated with 1 N NaOH released cumulatively highest total sugars consisting glucose + xylose after enzymatic hydrolysis compared to other pretreatment methods (*P* < 0.05).

### Ethanologenesis after SCB hydrolysis by *B. licheniformis* KU886221

SCB pretreated with 2% H_2_SO_4_, 3% H_3_PO_4_, 1% HCl, and 1 N NaOH were selected for ethanol production using *B. licheniformis* KU886221. The fermentation was carried out on FM2 medium modified by replacing SCB with selected pretreated SCB (100 g/L) following without/with enzymatic hydrolysis. When differently pretreated SCB without enzymatic hydrolysis were used, it was found that statistically higher ethanol was produced in which 1 N NaOH treated SCB was employed as carbon source in the fermentation medium (Table 1). Whereas, 32.89% more ethanol was produced when alkaline pretreated SCBH following enzymatic hydrolysis was employed as fuel’s feed (Table 2) compared to alkaline pretreated SCB without enzymatic hydrolysis. All experiments were performed in triplicates in 150 mL serum bottles under anaerobic conditions.

**Table 1:** Ethanol yield of *Bacillus licheniformis* KU886221 using differently pretreated SCB

Serial no.	Pretreatment	Incubation (h)	Total sugars (g/L)^a^	Ethanol (g/L)
1	2% H_2_SO_4_	0	41.227^b^ ± 0.430	0^i^
		24	36.556^cd^ ± 0.675	0.926^gh^ ± 0.229
		72	32.943^efg^ ± 1.009	2.103^cde^ ± 0.167
		120	31.434^fgh^ ± 0.570	2.61^abc^ ± 0.118
2	3% H_3_PO_4_	0	39.573^de^ ± 0.718	0^i^
		24	35.720^gh^ ± 0.325	1.126^fgh^ ± 0.117
		72	31.277^hi^ ± 0.657	2.44^bcd^ ± 0.092
		120	29.997^i^ ± 0.928	2.863^ab^ ± 0.154
3	1% HCl	0	44.964^a^ ± 0.527	0^i^
		24	39.427^bc^ ± 0.562	1.155^fgh^ ± 0.159
		72	34.533^def^ ± 0.595	2.06d^e^ ± 0.097
		120	32.317^fg^ ± 0.202	2.56^bcd^ ± 0.066
4	1 N NaOH	0	45.919^a^ ± 0.239	0^i^
		24	41.371^b^ ± 0.523	1.24^fgh^ ± 0.244
		72	35.687^de^ ± 0.603	2.793^ab^ ± 0.164
		120	31.800^fg^ ± 0.443	3.313^a^ ± 0.266
5	Control (untreated)	0	35.218^de^ ± 0.248	0^i^
		24	31.053^gh^ ± 0.335	0.69^h^ ± 0.039
		72	26.943^i^ ± 0.329	1.553^efg^ ± 0.073
		120	23.996^j^ ± 0.305	1.68^ef^ ± 0.037

^a^Glucose + xylose. Values signify mean ± SE of triplicates. Values that do not share an alphabet are significantly different from each other. Multi-factor analysis of variance (*P* < 0.05)

**Table 2:** Ethanol yield of *Bacillus licheniformis* KU886221 using SCB hydrolysate

Serial no.	Pretreatment	Incubation (h)	Total sugars (g/L)^a^	Ethanol (g/L)
1	2% H_2_SO_4_	0	41.952^bc^ ± 0.417	0^g^
		24	35.913^ef^ ± 0.676	1.101^f^ ± 0.124
		72	27.811^gh^ ± 0.333	3.491^d^ ± 0.129
		120	25.464^hi^ ± 0.579	4.294^bc^ ± 0.315
2	3% H_3_PO_3_	0	38.405^de^ ± 0.569	0^g^
		24	33.823^f^ ± 1.026	1.593^ef^ ± 0.143
		72	24.529^i^ ± 0.580	4.204^bc^ ± 0.047
		120	16.769^k^ ± 0.299	4.5413^ab^ ± 0.068
3	1% HCl	0	44.919^ab^ ± 0.212	0^g^
		24	41.214^cd^ ± 0.369	1.165^ef^ ± 0.179
		72	30.202^g^ ± 0.431	3.742^cd^ ± 0.080
		120	29.170^g^ ± 0.448	4.282^bc^ ± 0.053
4	1 N NaOH	0	46.306^a^ ± 0.465	0
		24	39.345^cd^ ± 0.126	1.483^ef^ ± 0.097
		72	28.981^g^ ± 0.306	3.955^bcd^ ± 0.163
		120	28.471 ^g^ ± 0.575	4.937^a^ ± 0.140
5	Control (untreated)	0	33.902^f^ ± 1.150	0^g^
		24	29.061^g^ ± 0.658	0.420^g^ ± 0.079
		72	25.139^hi^ ± 0.282	1.681^ef^ ± 0.066
		120	21.527^j^ ± 0.545	1.962^e^ ± 0.090

^a^Glucose + xylose. Values signify mean ± SE of triplicates. Values that do not share an alphabet are significantly different from each other. Multi-factor analysis of variance (*P* < 0.05)

### Optimization of enzymatic hydrolysis by central composite design

The optimum levels of Cellic Ctec 2 and Cellic Htec 2 Novozymes were obtained by solving the regression equation and analysis of the response surface contour plots. The global desirability function was also accomplished. The concentrations of Ctec and Htec Novozymes, ranging from 0.718 to 1.28 g/100 g of alkaline pretreated SCB and 0.118 to 0.68 g/100 g of alkaline pretreated SCB, respectively, were selected as independent variables (Table 3). The experimental design matrix is presented in Table 4 showing 11 experimental runs.

**Table 3:** Variables range in the central composite non-factorial experimental design

Variable	Ranges and levels				
	Axial −1.41	Minimum −1	Centre 0	Maximum 1	Axial 1.41
Ctec (g/100 g SCB)	0.718	0.8	1	1.2	1.28
Htec (g/100 g SCB)	0.118	0.2	0.4	0.6	0.68

**Table 4:** Central composite non-factorial (2^2^) experimental design matrix showing selected variables and their response result as sugars production

Standard run	Ctec (g/L)		Htec (g/L)		Total sugars (g/L)
	X_1_ g/100 g SCB	X_1_ coded value	X_2_ g/100 g SCB	X_2_ coded value	
1	0.8	−1	0.2	−1	31.07
2	0.8	−1	0.6	1	40.157
3	1.2	1	0.2	−1	39.8668
4	1.2	1	0.6	1	38.941
5	0.718	−1.41	0.4	0	34.458
6	1.28	1.41	0.4	0	41.109
7	1	0	0.118	−1.41	39.122
8	1	0	0.68	1.41	46.359
9 (C)	1	0	0.4	0	47.757
10 (C)	1	0	0.4	0	48.873
11 (C)	1	0	0.4	0	48.221

Analyzing the data from Tables 5 and 6, the model adequately adjusted to the experimental points, representing the confidence of the dependent variable analyzed. Considering the confidence level of 95%, the model obtained was significant, since the determination coefficient *R* ^2^ value was equal to 0.94 and adjusted *R* ^2^ was equal to 0.84. The *lack of fit* was insignificant (above 0.05) combined with the significant *F* and *P* values.

**Table 5:** Statistics for the regression of the optimization model for enzymatic hydrolysis using Ctec and Htec Novozymes for sugars liberation

	Regression	Standard error	Student’s *t*	*P*
Mean/interc.	−137.665	9.27795	−14.8378	0.004511
X_1_ (Ctec) (L)	302.033	16.85682	17.9176	0.003100
X_1_^2^ (Ctec) (Q)	−133.247	8.18171	−16.2860	0.003749
X_2_ (Htec) (g/L) (L)	130.403	11.72338	11.1234	0.007985
X_2_^2^ (Htec) (Q)	−70.464	8.18171	−8.6124	0.013215
1L by 2L	−62.580	9.63643	−6.4941	0.022900

**Table 6:** Analysis of variance (ANOVA) for enzymatic hydrolysis using Ctec and Htec Novozymes for sugars’ yield

	Sum of squares	Degree of freedom	Mean square	*F*	*P* value
Ctec (L)	35.0942	1	35.0942	59.0503	0.016516
Ctec (Q)	157.6309	1	157.6309	265.2338	0.003749
Htec (L)	41.6941	1	41.6941	70.1556	0.013956
Htec (Q)	44.0817	1	44.0817	74.1731	0.013215
1L by 2L	25.0640	1	25.0640	42.1734	0.022900
Lack of fit	14.9972	3	4.9991	8.4116	0.108106
Pure error	1.1886	2	0.5943		
Total SS	287.2233	10			

**Fig. 5:**
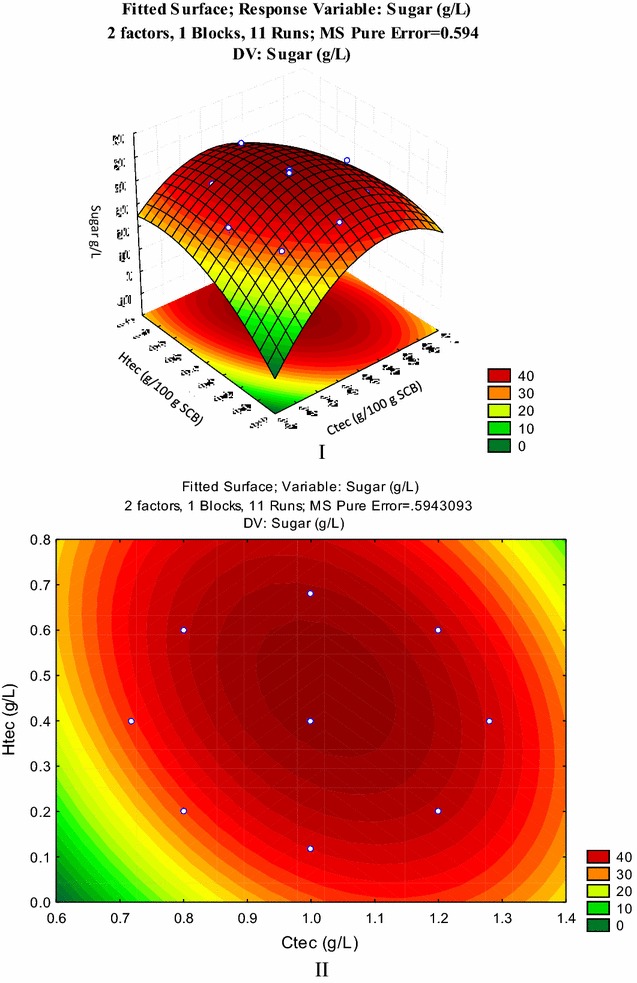
Response surface and contour plots indicating the effect of Ctec and Htec Novozymes on hydrolysis of alkaline pretreated sugarcane bagasse (**I**, **II**)

The response surface of the experimental design (Fig. 5) elucidates the effect of enzymatic hydrolysis on sugar liberation from sugarcane bagasse. The surfaces present the relation between dependent variables (Ctec and Htec enzymes) and independent factors (total sugars).

Global desirability value for optimized concentrations of dependent variables targeting for maximum production of response variable was 0.98, explaining the significance of the optimization by this method and justifying 98% of the maximum production of response variable (sugars). The optimal values of dependent variables (Ctec and Htec Novozymes) for sugar production from alkaline pretreated sugarcane bagasse were predicted from the desirability profile (see Additional file 1). It was proposed that by performing enzymatic hydrolysis with 1.024 g Ctec + 0.468 g Htec/100 g of alkaline pretreated SCB, 47.503 g total sugars should be released. The validation experiments were conducted in triplicates. The experimental value of sugars obtained was 46.69 g/100 g SCB with standard deviation of 0.675 that was within the confidence limits −95 and +95%, showing that these experimental findings were in full agreement with the model prediction (Table 7).

**Table 7:** Validation experiment analysis for hydrolysis of alkaline pretreated SCB for obtaining maximum quantity of simple sugars

Factor	Desirability	Limit %		Predicted value (g/L)	Experimental value (g/L)
		−95	+95		
Sugar (g/L)	0.984759	45.66	49.34526	47.503	46.69 ± 0.675

### Experimental design for optimization of fermentation using Taguchi orthogonal array

**Fig. 6:**
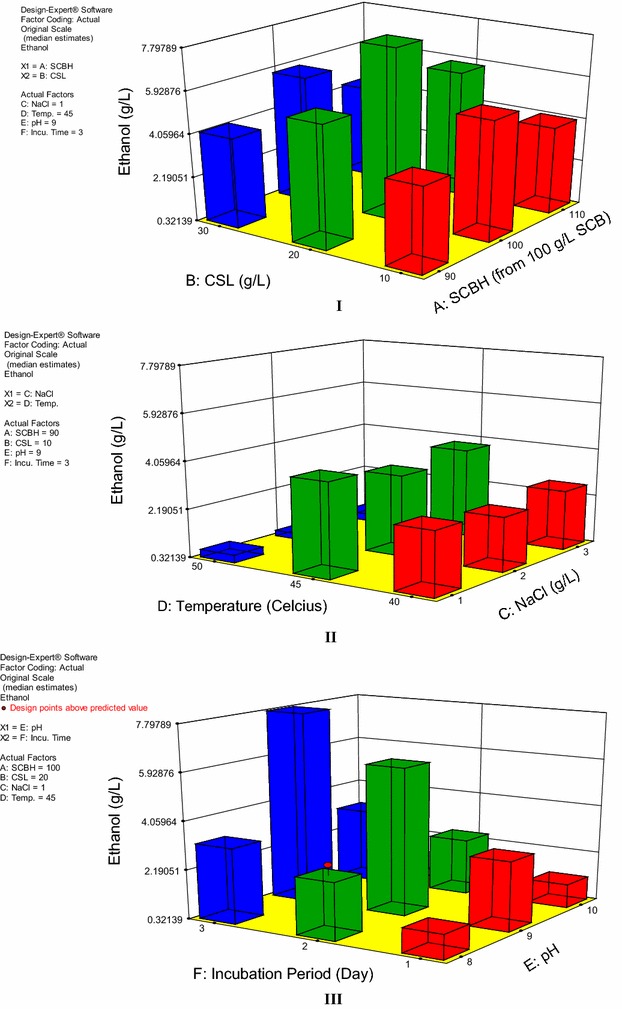
3-D surface model graph of Taguchi OA experimental design showing optimized levels of fermentation factors influencing ethanol production. **I** SCBH and CSL were set as variables; **II** incubation temperature and NaCl were set as variables; **III** incubation pH and incubation period were set as variables

Taguchi OA experimental design was incorporated for optimization of ethanol fermentation parameters using *B. licheniformis* KU886221. The three levels of six fermentation factors affecting the ethanol fermentation are described in Table 8, whereas the 27 runs Taguchi OA experimental design along with the results are shown in Table 9. The results show that the most suitable factors for ethanol fermentation are SCBH derived from 100 g/L alkaline pretreated SCB, 20 g/L CSL, 1 g/L sodium chloride (NaCl), 45 °C incubation temperature, incubation pH 9, and 5 days of incubation time. 3-D surface model graph of Taguchi OA experimental design shows the optimized levels of fermentation factors influencing ethanol production (Fig. 6).

**Table 8:** Selected three levels of fermentation factors for ethanol production from *Bacillus licheniformis* KU886221 using Taguchi OA experimental design

Serial no.	Factor	Name	Units	Low level	Level	High level
1	A	SCBH	g/L	90	100	110
2	B	CSL	g/L	10	20	30
3	C	NaCl	g/L	1	2	3
4	D	Temp.	C	40	45	50
5	E	pH	0–14	8	9	10
6	F	Incu. time	Day	1	2	3

**Table 9:** Taguchi experimental design for optimization of fermentation parameters for ethanol production from *Bacillus licheniformis* KU886221

Run	SCBH (g/L)	CSL (g/L)	NaCl (g/L)	Temp (°C)	pH	Incubation (days)	Ethanol (g/L)
1	100	10	3	45	10	1	0.547
2	100	20	1	45	8	2	2.938
3	90	30	3	40	10	3	0.661
4	90	30	3	45	8	1	1.650
5	90	10	1	50	10	3	0.815
6	100	10	3	40	9	3	3.214
7	90	20	2	45	10	3	0.555
8	100	10	3	50	8	2	1.106
9	90	10	1	45	9	2	3.953
10	100	30	2	40	8	2	2.146
11	100	30	2	45	9	3	4.808
12	90	30	3	50	9	2	0.471
13	110	10	2	45	8	3	4.225
14	110	20	3	45	9	1	2.678
15	110	10	2	50	9	1	0.204
16	110	10	2	40	10	2	0.362
17	90	20	2	40	9	2	3.143
18	100	20	1	40	10	1	0.840
19	110	30	1	45	10	2	1.779
20	90	20	2	50	8	1	0.086
21	110	20	3	50	10	2	0.107
22	110	30	1	50	8	3	0.434
23	90	10	1	40	8	1	1.025
24	100	20	1	50	9	3	0.854
25	110	20	3	40	8	3	3.596
26	100	30	2	50	10	1	0.106
27	110	30	1	40	9	1	1.359

Results of the 27 runs for the experimental design executed revealed that the maximum production of ethanol was expected to be 7.79 g/L with standard deviation 0.446 under the optimized conditions of fermentation factors. However, under these selected fermentation conditions, the ethanol production appeared 7.612 g/L with standard deviation of 0.178 (while performing experiment in triplicates). Since the variance between the predicted and actual production was 2.28%, the result was considered as acceptable.

Analysis of variance (ANOVA) of the experimental design’s results revealed that changing of incubation temperature and pH are the most important factors affecting ethanol fermentation (Table 10). Considering the confidence level of 99%, the model obtained was significant, since the coefficient *R*-square (*R* ^2^) value was equal to 0.96 and adjusted *R* ^2^ was equal to 0.855. The Model *F* value of 8.69 implies that the model is significant. There is only a 0.65% chance that a “Model *F* value” of this large could occur due to noise.

**Table 10:** Results of analysis of variance (ANOVA) of the Taguchi OA experimental design

Source	Sum of squares	Degree of freedom	Mean square	*F* value	*P* value Prob > *F*
Model	34.76	20	1.74	8.69	0.0065
A-SCBH	0.55	2	0.28	1.39	0.3197
B-CSL	0.19	2	0.095	0.48	0.6432
C-NaCl	1.39	2	0.69	3.46	0.0999
D-temp.	17.42	2	8.71	43.57	0.0003
E-pH	7.5	2	3.75	18.75	0.0026
F-incubation time	4.32	2	2.16	10.8	0.0103
BE	0.74	4	0.19	0.93	0.506
CE	2.65	4	0.66	3.31	0.0929
Residual	1.2	6	0.2		
Cor total	35.96	26			

### Bench-scale ethanol fermentation employing *B. licheniformis* KU886221

#### Fermentation in bioreactor

**Fig. 7:**
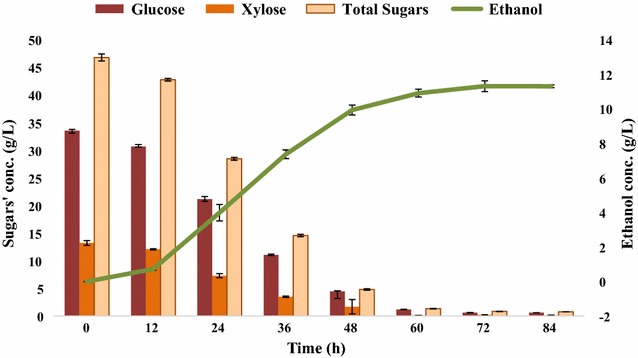
Batch fermentation of SCBH by *Bacillus licheniformis* KU886221 for ethanol production. *Error bars* represent mean ± SEM

In batch fermentation of *B. licheniformis* KU886221 in stirred-tank bioreactor for ethanol production, maximum productivity of 0.205 g/L h was obtained at 36 h of fermentation, whereas overall yield of batch fermentation was 0.245 g of ethanol/g of sugars (glucose + xylose) consumed which corresponds to 0.909 mol of ethanol/mol of sugars consumed (Fig. 7). The percentage reduction of substrate (PRS) was 98.4 which is significant amount of substrate consumption into product. In terms of biomass, 0.114 g ethanol/g of alkaline pretreated SCB was produced. Cell density was noted by measuring the optical density of the cell suspension during batch fermentation. The bacterial growth ranged from 0.168 to 1.86 O.D. at 12 and 60 h postinoculations, respectively.

**Fig. 8:**
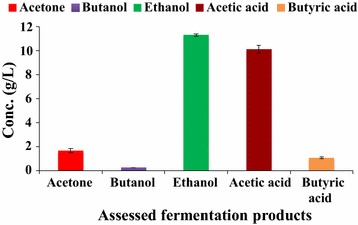
Fermentation kinetics of batch fermentation from SCBH by *Bacillus licheniformis* KU886221 at 84 h. *Error bars* represent mean ± SEM

Figure 8 presents the results of fermentation kinetics of batch fermentation in bioreactor at the end of 84 h of fermentation. It shows that along with ethanol, some acetone and butanol were also produced, whereas lesser amount of acetic acid was also produced from ethanol due to which pH of the medium decreased.

##### Fed-batch fermentation

**Fig. 9:**
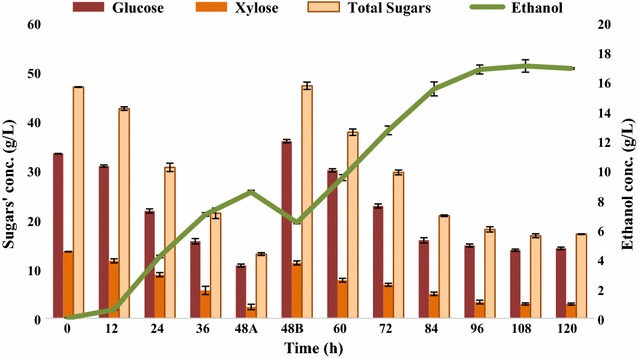
Fed-batch fermentation of SCBH by *Bacillus licheniformis* KU886221 for ethanol production. 48A-before addition of second feed and 48B: following second feed 5× (200 mL). *Error bars* represent mean ± SEM

The results of fed-batch fermentation showed that when substrate addition was made, the overall substrate consumption increased showing that the fermentative microbes consumed more substrate and produced more product (Fig. 9). However, after attaining a certain level of ethanol or toxic metabolites, the fermentative microbes stopped producing ethanol despite the presence of substrate in the fermentation medium. The ethanol yield was found to be 0.264 g/g of sugars consumed which corresponded to 0.973 mol of ethanol/mol of sugars consumed. In terms of biomass, 0.123 g ethanol/g of alkaline pretreated SCB was produced. The bacterial growth ranged from 0.164 to 1.925 O.D., at 12- and 96-h postinoculations, respectively.

##### Immobilized-cell fermentation using fibrous-bed bioreactor (FBB)

**Fig. 10:**
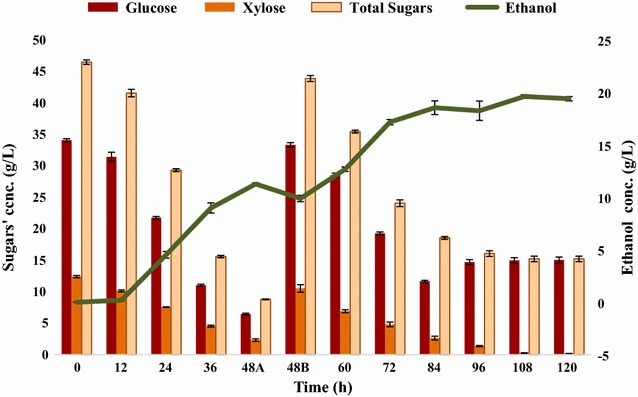
Fed-batch fermentation of SCBH involving FBB by *Bacillus licheniformis* KU886221for ethanol production. 48A: before addition of second feed and 48B: following second feed 5× (200 mL) addition. *Error bars* represent mean ± SEM

**Fig. 11:**
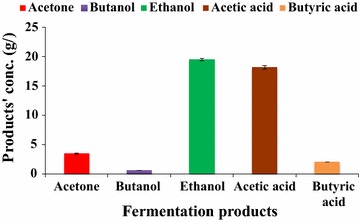
Fermentation kinetics of *Bacillus licheniformis* KU886221 at 120 h of fed-batch fermentation involving FBB. *Error bars* represent mean ± SEM

The results of fed-batch fermentation involving FBB (Fig. 10) indicate that PRS was increased from 78.94 to 85.031 due to FBB attachment for fed-batch fermentation setup. The ethanol yield was 0.279 g/g of sugars consumed which corresponds to 1.07 mol of ethanol/mol of sugars consumed. In terms of biomass, 0.131 g of ethanol/g of alkaline pretreated SCB was produced. The bacterial growth ranged from 0.432 to 1.353 O.D. at 12- and 72-h postinoculations, respectively. The fermentation kinetics of the immobilized-cell fermentation using FBB was studied at the end of fermentation (Fig. 11).

At the end of fed-batch fermentation involving FBB, the downstream product recovery was done though gas stripping. After 3 h of gas stripping, a total of 158 mL of condensate containing 56.88 g/L ethanol, 15.18 g/L acetone, and 20.02 g/L butanol were obtained.

##### Effect of ethanol removal on fermentation

**Fig. 12:**
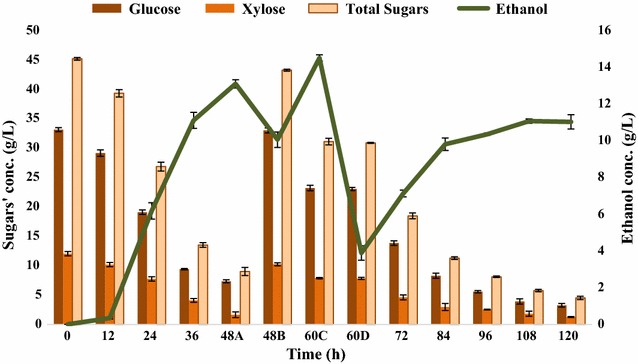
Fed-batch fermentation involving FBB and gas tripping by *Bacillus licheniformis* KU886221 for ethanol production. 48A: before addition of second feed, 48B: following second feed 5× (200 mL) addition, 60C: gas stripping and 60D: after gas stripping. *Error bars* represent mean ± SEM

Next experiment elucidates the effect of partial ethanol removal during fed-batch fermentation involving FBB on the ethanol fermentation (Fig. 12). The gas stripping was done at 60 h of fermentation when the ethanol titer was above 10 g/L. Significant increase in ethanol production from 19.389 to 22.97 g/L was observed by removing the ethanol during fermentation which corresponded to 1.1407 mol of ethanol per mole of sugars consumed. In terms of biomass, 0.135 g of ethanol/g of alkaline pretreated SCB was produced. The bacterial growth ranged from 0.236 to 1.788 O.D. at 12- and 96-h postinoculation, respectively. In addition, significantly greater amount of condensate (260 mL) was obtained as a result of 6 h of gas stripping containing 58.1 g/L ethanol, 16.9 g/L acetone, and 21.6 g/L butanol.

##### Bioethanol production under non-aseptic conditions

**Fig. 13:**
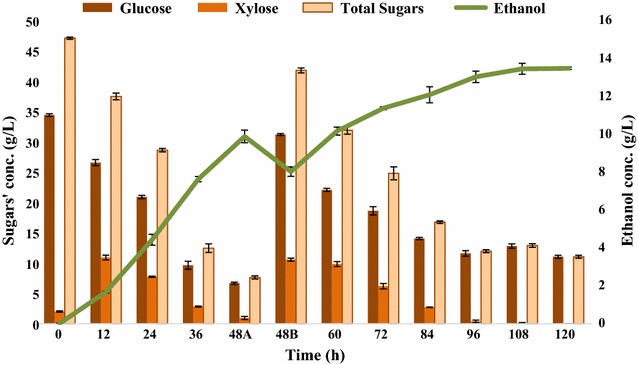
Fed-batch fermentation of SCBH involving FBB by *Bacillus licheniformis* KU886221 for ethanol production under non-aseptic conditions. 48A: before addition of second feed and 48B: following second feed 5× (200 mL) added. *Error bars* represent mean ± SEM

**Fig. 14:**
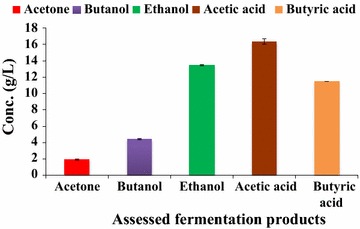
Fermentation kinetics of *Bacillus licheniformis* KU886221 (at 120 h) of fed-batch fermentation involving FBB under non-aseptic conditions. *Error bars* represent mean ± SEM

The fed-batch fermentation involving FBB was studied under non-aseptic fermentation for ethanol production (Figs. 13, 14). In terms of biomass, 0.089 g of ethanol/g of alkaline pretreated SCB was produced. The bacterial growth ranged from 0.218 to 2.459 O.D. at 12 and 96 h post inoculations, respectively. Overall yield decreased compared to aseptic fermentation conditions from 0.279 g of ethanol/g of substrate to 0.192 which corresponds to 0.5703 mol ethanol per mole of sugar consumed. At the end of non-aseptic fermentations by *B. licheniformis* KU886221, low quantity of condensate (120 mL) was obtained following gas stripping containing 51.12 g/L ethanol, 6.79 g/L acetone, and 32.08 g/L butanol.

## Discussion

Principal aim of this study was to extract the energy from renewable waste sugarcane bagasse under non-aseptic extreme conditions in the form of bioethanol. For this purpose, alkali-thermophilic ethanologens were isolated from the soil sampled from the vicinity of hot water effluent near Balkasar oil refinery, Chakwal, Pakistan. The mud temperature was 50–55 °C with pH ranging 9–10 at the sampling time. Such habitats are favorable conditions for isolation of alkali-thermophiles [26]. Further valuation of cellulolytic/ethanologenic potential of the isolates was done by replacing glucose with sugarcane bagasse as substrate in the fermentation medium. On the basis of higher ethanol production, isolate ML-07 was selected, pure-cultured, and identified through 16S rRNA gene sequencing, and was recognized as a cellulolytic and mild alkali-thermophilic strain of *B. licheniformis.* Consequently, it was allotted the accession no. KU886221. It was previously reported that some strains of *B. licheniformis* were found to produce ethanol in fermentation medium [27].

As mentioned before, SCB was selected as substrate for ethanol fermentation. Composition of sugarcane bagasse was 31.168% cellulose, 21.048% xylan, and 23.633% lignin. Many thermophilic bacteria capable of utilizing hexoses/pentoses from cellulose/hemicellulose, respectively, directly/indirectly are considered promising for ethanol fermentation [16, 28–30]. For that reason, it can be speculated that both cellulose and hemicellulose are valuable carbon substrates for thermophilic ethanol fermentation.

Cellulose is generally recalcitrant to hydrolysis; therefore, ethanol production from lignocellulosic biomass requires separation of its fractions through pretreatment methods [31]. The chemicals used in pretreatment expose the crystalline cellulose and enhance its porosity by breaking down lignin protection around it, which, in turn, facilitates polysaccharide hydrolysis [32, 33]. For this purpose, optimum pretreatment concentrations of H_2_SO_4_, H_3_PO_4_, HCl, and NaOH for SCB hydrolysis were explored. The outcomes indicated that the alkaline pretreatment with 1 N sodium hydroxide of SCB significantly reduced lignin contents and produced cumulatively greater sugar contents (glucose and xylose) after enzymatic hydrolysis as presented in Additional file 2. This is analogous to the findings of previously reported research indicating improved performance in sugar yield after enzymatic hydrolysis of alkaline pretreated SCB, delignification, and biomass utilization, compared to acid pretreatment [33, 34].

When utilization potential of differently pretreated SCB for ethanol production was evaluated, it appeared that ethanol production from *B. licheniformis* KU886221 was also greater for alkaline pretreated SCB possibly due to less inhibitory compounds’ production compared to acid pretreatments that produce more inhibitory compounds for subsequent fermentation processes as reported previously [35]. This is parallel to the previous finding (see Additional file 2) presenting that microbe *Saccharomyces cerevisiae* was able to yield significantly greater ethanol (0.39 g/g sugar) employing timothy grass hydrolysate containing 57.4 g/L sugar compared to the ethanol yield (0.36 g/g sugar) employing pine wood hydrolysate containing 68.5 g/L sugar and wheat straw hydrolysate’s ethanol yield (0.35 g/g hexose) from 63.6 g/L sugar [36].

In subsequent experiment, ethanol production was evaluated employing pretreated SCB following enzymatic hydrolysis (SCBH). It was found that ethanol production increased 32.89% for SCBH. Furthermore, when the bacterium was analyzed to ferment glucose, xylose, and cellulose separately in fermentation medium FM4, it was revealed that after 72 h of incubation, glucose was the preferred sugar substrate compared to cellulose. It also consumed significant amounts of xylose and mannose. Approximately 60% reduced ethanol was produced using cellulose directly as substrate in comparison with glucose. Thence, alkaline pretreated SCB hydrolysate was selected as the carbon source in fermentation medium for further investigation.

RSM is an assemblage of statistical performances for scheming experimentations, constructing models, valuing the special effects of experimental factors, and scrutinizing for the optimum conditions facilitating the whole phenomenon. Enzymatic hydrolysis was performed by a combination of two enzymes, i.e., cellulase (Cellic Ctec-2 Novozyme) and hemicellulase (Htec Novozymes) for breaking down biomass to maximum amounts of fermentable sugars. The effect of Ctec and Htec enzymes’ quantities on consequent alkaline pretreated SCB hydrolysis for attaining simple fermentable sugars was studied by central composite design.

A quadratic (second-order) polynomial equation was proposed including all interaction terms relating independent and response variables:

$${{Y }} = \, \beta_{0} + \beta_{1} {{X}}_{1}^{2} + \beta_{2} {{X}}_{2}^{2} + \beta_{3} {{X}}_{1} + \beta_{4} {{X}}_{2} + {{X}}_{1} {{AX}}_{2} + \varepsilon.$$

The results of the second-order response surface models for the sugar hydrolysis in the form of regression and analysis of variance (ANOVA) were studied under 95% of confidence interval (*P* ≤ 0.05) and pure error. Desirability profiling with the help of Statistica software enabled to predict enzymes’ optimum quantities. After validation of the experiment, enzymes optimum dose was determined as 1.024 g Cellic Ctec Novozymes + 0.468 g Htec Novozymes/100 g of alkaline pretreated SCB that was found to be compliant to the previously investigated [37].

The purpose of the present investigation was to exploit the low-cost substrate for the production of clean and environmentally friendly biofuels. In this regard, corn steep liquor (CSL), a chief by-product of corn starch processing, was used as low-cost nitrogen source. It contains significant amount of proteins, amino acids, vitamins, minerals, and trace elements, and can suitably replace yeast extract and peptone in alcoholic fermentations [38–42]. Specifically, CSL has been reported effective for ethanol fermentations previously [39, 40, 43–45]. In advance experiment, potential of CSL as complex nitrogen source for ethanol production substituting peptone and yeast extract in the fermentation medium was evaluated. It was found that 3.24% more ethanol was produced when CSL was used in comparison with peptone + yeast extract in the fermentation medium. This increased ethanol production was possibly due to the complex nutritious nature of CSL [38, 40].

Concentration of alkaline pretreated SCB hydrolysate, CSL and NaCl, incubation temperature, pH, and fermentation period were optimized for ethanol fermentation using *B. licheniformis* KU886221. Experimentation assisted by Taguchi OA experimental design using the Design Expert 8 software enabled us to infer that changing of incubation temperature (optimum: 45 °C) and alkaline pH (optimum: 9) are most significant factors affecting ethanol fermentation. Furthermore, a batch culture was carried out under optimized conditions of ethanol fermentation in bench-scale stirred-tank bioreactor. Ethanol yield of 0.909 mol of ethanol/mol of sugars consumed was obtained after 84 h of incubation, corresponding to 0.279 g ethanol/g of sugars consumed. Though reduced ethanol yield in comparison with the yield by mesophilic yeast, *S. cerevisiae* (as shown in Additional file 2) was obtained, but the use of thermophilic fermentative bacteria is considered more advantageous where LCB is used as fuels’ feed as unlike most mesophilic yeasts, the thermophilic fermentative bacteria are able to ferment hexoses as well as pentoses to ethanol [36, 46]. The percent reduction of substrate was 98.5 showing significant conversion of substrate into product with ethanol titer of 11.301 g/L. In terms of biomass, 0.114 g ethanol/g of alkaline pretreated SCB was yielded. It is noticeable that the slope for ethanol production regarding sugar consumption was nearly constant, signifying that this integrated fermentation was very steady and reliable with ethanol as the primarily chief product.

Next, the fed-batch fermentation was performed to understand the influence of substrate addition on batch fermentation. The overall ethanol titer was 16.896 g/L associated with 78.94% substrate consumption. In terms of biomass, 0.123 g ethanol/g of alkaline pretreated SCB was obtained at the end of fed-batch fermentation experiment. The molar ethanol yield was 0.973 mol ethanol/mol sugars consumed. This lower substrate consumption was possibly due to accumulation of inhibitory fermentation metabolites including ethanol.

To lessen the toxic effect of higher density of cells on their further growth/fermentative potential, further adaptation in fed-batch bioreactor was applied by attaching fibrous-bed bioreactor (FBB) to the fermentor [22, 47]. It is also very innovative fermentation approach to improve solvent tolerance facilitating ethanol fermentation. Increased ethanol production with 85.031% substrate utilization was observed with 1.076 mol ethanol/mol of sugars consumed. In terms of biomass, 0.131 g ethanol/g of alkaline pretreated SCB was achieved at the end of fermentation. This revealed that immobilized-cell fermentation might be capable of enhancing ethanol production by trapping the cells in fibrous-bed, thus facilitating increased cell tolerance to toxic fermentation metabolites and increase cell viability inside main bioreactor. Ethanol titer was significantly increased from 16.896 to 19.39 g/L with the attachment of FBB.

To investigate the ethanol-induced inhibition, further study was done by partially removing ethanol by in situ gas stripping from the fermentation medium. Gas stripping is an established product recovery technique for ABE separation [23, 48–51]. It was confirmed that ethanol intolerance was one of the reasons of growth inhibition. Ethanol titer of 21.637 g/L (1.1406 mol ethanol/mol of sugars consumed) was observed with 94.295% substrate consumption. Results are compliant with the previous findings that removal of inhibitory compounds leads to a better performance in sugar conversion to products [48, 52]. Thus, gas stripping is proved to be an effective separation technique used to apprehend synchronized ethanol recovery and overcome product repression due to ethanol-induced inhibition [52, 53]. In terms of biomass, 0.135 g ethanol/g of alkaline pretreated SCB was obtained at the end of fed-batch fermentation involving FBB and gas stripping.

Finally, fed-batch fermentation involving FBB was studied under non-aseptic conditions. The results indicated that 13.466 g/L ethanol corresponding 0.570 mol ethanol/mol of sugars consumed was produced at the end of fermentation (at 120 h). In terms of biomass, 0.089 g ethanol/g of alkaline pretreated SCB was obtained. Comparing aseptic conditions, 30.5% reduced ethanol yield was obtained under non-aseptic conditions showing the likelihood of some contaminant in the fermentation culture. However, cost of sterilization is one of the major obstacles for developing lignocellulosic bio-refineries. Therefore, even a reduced product yield might be considered appealing while saving the cost of sterilization.

In the present study, besides ABE, significant amounts of acetic acid and some butyric acid were also produced during fermentation. ABE were also formed in the condensate after gas stripping. Realizing separation of ABE along with acetic acid and butyric acid would make the fermentation methodology more efficient as these by-products are very profitable.

## Conclusion

Purpose of the present investigation was to exploit the locally available lignocellulosic waste for the production of clean and environment friendly energy fuel bioethanol employing extremophiles. In this regard, the bioethanol production by alkali-thermophilic fermentative microbes from bio-waste such as sugarcane bagasse would not only be sustainable, but the extreme conditions will employ a bioprocess for high selection pressure for reducing the likelihood of microbial contaminations. Thus, it will be possible to maintain aseptic environment without spending much for sterilization. Successful exploitation of low-cost substrate for biofuels’ production under moderate alkali-thermophilic conditions appeared promising for the development of large-scale bio-fermentation processes. For this purpose, it is extremely important to explore the locally available fermentative ethanologenic thermophiles and understand their metabolic requirements while utilizing lignocellulosic biomass. It is foreseeable that understanding of non-aseptic extremophilic fermentations utilizing agro-industrial waste as necessitated in the present investigation for development of large-scale cost-effective, eco-friendly biofuel generation processes will pave the way to achieve one of the greatest benefits of mankind.

## Additional files

**Additional file 1: Figure S1.** Desirability profile for predicted values of Ctec and Htec Novozymes for sugarcane bagasse hydrolysis.

**Additional file 2: Table S1.** Comparison of sugar hydrolysis/ethanol fermentation employing different LCB.

## Abbreviations

LCB: lignocellulosic biomass
SCB: sugarcane bagasse
SCBH: sugarcane bagasse hydrolysate
CSL: corn steep liquor
FBB: fibrous-bed bioreactor
PRS: percentage reduction of substrate
CCD: central composite design
RSM: response surface methodology
