# Supplementary material for: Moderate alkali-thermophilic ethanologenesis by locally isolated Bacillus licheniformis from Pakistan employing sugarcane bagasse: a comparative aspect of aseptic and non-aseptic fermentations
Source: Biotechnol Biofuels. 2017 Apr 24;10:105. doi: 10.1186/s13068-017-0785-1 (PMC5402650; doi:10.1186/s13068-017-0785-1)
Supplement: Supplementary file 1 — Additional file 1: Figure S1. Desirability profile for predicted values of Ctec and Htec Novozymes for sugarcane bagasse hydrolysis. [file 13068_2017_785_MOESM1_ESM.docx]

**Fig S1**. Desirability profile for predicted values of Ctec and Htec Novozymes for sugarcane bagasse hydrolysis
