# Supplementary material for: Moderate alkali-thermophilic ethanologenesis by locally isolated Bacillus licheniformis from Pakistan employing sugarcane bagasse: a comparative aspect of aseptic and non-aseptic fermentations
Source: Biotechnol Biofuels. 2017 Apr 24;10:105. doi: 10.1186/s13068-017-0785-1 (PMC5402650; doi:10.1186/s13068-017-0785-1)
Supplement: Supplementary file 2 — Additional file 2: Table S1. Comparison of sugar hydrolysis/ethanol fermentation employing different LCB. [file 13068_2017_785_MOESM2_ESM.docx]

**Table S1: Comparison of sugar hydrolysis/ethanol fermentation employing different LCB**

| **LCB** | **Pretreatment Chemical** | **Hydrolyzing Enzyme (Brand name)** | **Sugar yield**  **(glucose + xylose**  **(g/L)** | **Fermentative microbe** | **Average ethanol yield** | **References** |
| --- | --- | --- | --- | --- | --- | --- |
| Pine wood | 2 % H_2_ SO_4_ | Celluclast 1.5L + Novozyme 188 + Xylanase 1 | 68.5 | S. cerevisiae ATCC | 0.36 g/g glucose | 36 |
| Timothy grass | 1.5 % H_2_ SO_4_ |  | 57.4 |  | 0.39 g/g glucose | 36 |
| Wheat Straw | 1.5 % H_2_ SO_4_ |  | 63.6 |  | 0.35 g/g glucose | 36 |
| Sugarcane bagasse | 1N NaOH | NS22074 NS50010 Novozymes | 41.4 | - | - | 33 |
| Sugarcane bagasse | 2 % H_2_ SO_4_ + 4 % NaOH | NS22074 NS50010 Novozymes | 23.28 | - | - | 33 |
